# Nematicidal activity of *Annona crassiflora* leaf extract on *Caenorhabditis elegans*

**DOI:** 10.1186/s13071-015-0708-6

**Published:** 2015-02-19

**Authors:** Alan Rodrigues Teixeira Machado, Sebastião Rodrigo Ferreira, Felipe da Silva Medeiros, Ricardo Toshio Fujiwara, José Dias de Souza Filho, Lúcia Pinheiro Santos Pimenta

**Affiliations:** Departamento de Química, Instituto de Ciências Exatas, Universidade Federal de Minas Gerais, Av. Antônio Carlos 6627, Belo Horizonte, MG 31270-901 Brazil; Departamento de Parasitologia, Instituto de Ciências Biológicas, Universidade Federal de Minas Gerais, Av. Antônio Carlos, 6627, Pampulha, CEP 31270-901 Belo Horizonte, Minas Gerais Brazil

**Keywords:** *Annona crassiflora*, *Caenorhabditis elegans*, Metabolite, Nematicidal activity

## Abstract

**Background:**

The aim of this work was to investigate the potential nematicidal activity of *Annona crassiflora* leaf extract against *Caenorhabditis elegans*.

**Methods:**

The hydroalcoholic leaf extract and its fractions (dichloromethane, ethyl acetate, methanol and water) were submitted to mobility assay against the roundworm *Caenorhabditis elegans*. GC-MS and NMR analysis were performed in order to identify metabolites.

**Results:**

The dichloromethane and ethyl acetate fractions showed to be the most active among the hydroalcoholic leaf extracts and its four fractions. The percentages of *C. elegans* larvae immobility were 98.13 and 89.66%, respectively, at a concentration of 1000 μg.mL^−1^. Besides some amino acids, palmitic acid methyl ester, 2-isopropyl-5-methylcyclohexanol, oleic acid methyl esther, stearic acid methyl ester, quercetin and kaempferol were also identified in these fractions.

**Conclusion:**

The results indicated that of *A. crassiflora* leaf ethanolic extract has a good potential as a source for natural nematicide.

## Background

Parasitism attributed to nematodes is a worldwide problem that can negatively affect livestock, human health and plant growth. In animal breeding it can result in stunted growth, lower herd weight gain and can negatively influence meat quality. Sometimes, depending on the severity of the infection, it may lead to animal death [1,2]. Regarding plant production, nematodes are highlighted for causing major economic losses to agriculture [3]. The most widespread form of nematode control is that performed with synthetic anthelmintics. However, their improper use has favored the selection of resistant strains, besides being toxic and impactful to the environment [4]. In the last decade there has been an increasing search for new methods of controlling animal and plant parasitic nematodes, using methods less toxic to the environment and to people [3]. Among these, the search for compounds from natural sources stands out.

Diverse genera of plants have shown their potential as sources of various metabolites to be used in nematode control [3]. Among these, the genus *Annona* may be cited because the extracts of different species of this genus have shown nematicidal activity against various nematodes, such as *Haemonchus* sp., *Bursaphelenchus* sp. and *Meloidogyne* sp. [5-7]. *Annona crassiflora* Mart is a native tree from the Brazilian Cerrado, which presents various bioactive compounds [8,9]. However, its nematicidal potential has not been investigated. Therefore, the present study aimed to evaluate the *in vitro* activity of the extracts of *A. crassiflora* on the free-living nematode *Caenorhabditis elegans* as a model, to identify metabolites of *A. crassiflora* with nematicidal properties which can be of great value for the development of new products for nematode control.

## Methods

### General experimental procedures

The NMR spectra (one - and two-dimensional) were obtained in Bruker *AVANCE* DRX400 spectrometer. The solvents and reagents used were analytical grade. Column chromatography (CC) was performed on silica gel 70–230 μm from Sigma-Aldrich. TLC was performed on SiO_2_ plates (Sigma-Aldrich, 0.25 mm) with visualization under UV lamp (254 and 365 nm) and vanillin/sulfuric acid as spray reagent.

### Plant material

*Annona crassiflora* leaves were collected in Itatiaiuçu, Minas Gerais, Brazil, in July 2007. A voucher specimen was deposited at the Instituto de Ciências Biológicas Herbarium (BHCB, n° 22.988), Universidade Federal de Minas Gerais.

### Extraction and characterization of metabolites by NMR spectroscopy

According to the methodology described by Kim *et al*., [10], 50 mg of dried, ground leaves underwent extraction with a combination of 0.75 mL of KH_2_PO_4_ buffer solution in D_2_O (90 mM, pH = 6.0) containing 0.0 1% trimethylsilylpropionic acid (TSP-*d*_4_) as internal reference and 0.75 mL methanol-*d*_4_. After solvent addition, samples were vortexed for 1 minute at room temperature, followed by sonication for 20 min and centrifugation at 10968 × *g* for 15 min. 800 μL of supernatant was transferred to 5 mm NMR tubes. Identification of metabolites signals on the ^1^H NMR spectra was carried out comparing the signals observed in the hydrogen spectrum with the reported ^1^H NMR signals of compounds available in the literature obtained under the same condition [11-14].

The ^1^H-^1^H *J*-resolved spectrum was acquired using ns 8, tdF1 256, tdF2 1 K sw F2 4.0 kHz, swF1 80 Hz and d1 1.0 s. Datasets were zero filled to 1096 points in F1 and both dimensions were multiplied by sine-bell function (SSB = 0) prior to double complex Fourier transform. The *J*-resolved spectra were tilted by 45°, symmetrized about F1, and calibrated to TMS, using topspin (version 3.1, Bruker). The ^1^H-^1^H COSY experiment was acquired with a 1.0 s relaxation delay and 8.0 kHz spectral width in both dimensions.

### Extraction and isolation of metabolites

The dried and ground leaves (500 g) were subjected to exhaustive extraction by percolation with hexane at room temperature, followed by hydroalcoholic solution (CH_3_CH_2_OH: H_2_O, 8/2). The extracts were concentrated under reduced pressure to obtain the crude hexane extract (2.3% w/w) and the crude hydroalcoholic extract (35.2% w/w). Approximately 10 g of hydroalcoholic extract was suspended in water and partitioned with solvents of increasing polarity, obtaining, after solvent removal, the following fractions and their yields: dichloromethane (1.7% w/w), ethyl acetate (1.5% w/w), methanol (89.7% w/w) and water (3.2% w/w). All fractions were tested for nematicidal activity for selection of the material to be purified. Those that showed better results were subjected to fractionation on silica gel column chromatography eluted by step gradient with hexane/ ethyl acetate from 5% to 100%.

### GC-MS analysis

The GC-MS analyses were performed on gas chromatography-mass spectrometer Shimadzu GCMS-QP2010 Plus, equipped with an AOC-10 automatic injection system. The analyses were performed using a Rxi-1 capillary column (30 m × 0.25 mm, coated with100% polydimethylsiloxane 0.25 μm film thickness) and He (helium) as carrier gas (flow rate 7.2 ml/min). The injector temperature was 250°C, and the temperature program ranged from 150°C to 300°C at 3°C/min. The injected volume was 1 μL, in split-mode at a ratio of 10:1. MS analysis was carried out in quadrupole MS system (QP-2010plus) operating at 70 eV under the same conditions as described above. The identification of the compounds was performed by comparison with mass spectral data from NIST 62 and NIST 12 libraries.

### Methyl esters synthesis

150 mL round bottom flask equipped with reflux condenser was charged with palmitic acid (1 g), hexane (20 mL) and methanol containing 2,0% (v/v) concentrated H_2_SO_4_. The reaction mixture was stirred and refluxed for 1 h. The progress of the reaction was monitored by TLC, CG-MS and ^1^H NMR. After the reaction was complete, the reaction mixture was washed with aqueous sodium chloride 10% (w/v). The organic layer was removed, dried over anhydrous Na_2_SO_4_ and concentrated under vacuum. The same procedure was repeated with myristic acid and stearic acid.

### *C. elegans* production

The strain of *C. elegans* used in the experiment was kindly provided by Universidade de São Paulo (USP). L3 larvae of *C. elegans* were grown on 8P NGM plates according to the methodology previously described [15,16]. After seven days of culture in a BOD incubator at 20°C, the plates were washed with M9 medium (Stiernagle 2006) and filtered through three sieves with 40 μm, 30 μm and 20 μm pores. L3 larvae retained in the 20 μm strainer were collected by backwashing. The obtained larvae were washed by centrifugation at 700 g for 4 minutes, followed by two washes with M9 medium. The average size of these larvae was 527 μ (σ 3.4) long by 23.3 μ in diameter (σ 1.9).

### Nematicidal assay against *C. elegans*

The *C. elegans* L3 were resuspended in M9 and approximately 1000 larvae in 100 μL of suspension were added to each well in a 96 wells micro plate. Tested extracts and substances were dissolved in 1.0 mL of an aqueous 1% (v/v) DMSO, were then added at the concentrations 0.01; 0.1; 1; 10; 100 and 1000 μg.mL^−1^. Plates containing extracts or substances and larvae were stored in BOD incubator at 20°C. After 72 hours, 10 μL of solution containing approximately 100 larvae was removed from each well for analysis and quantification of paralyzed larvae number was carried out using an optical microscope at 100× magnification. Larvae were considered paralyzed when presenting straight body and absence of any mobility.

### Statistical analyses

Values were submitted to analysis of variance (ANOVA), followed by means separation using the Scott-Knott test (*P* ≤ 0.05). For this purpose, the software SISVAR (Sistema para análises Estatísticas, Versão 5.1, UFLA, Lavras, 2006). Nonlinear regression analysis was used to calculate the ED_50_ value utilized of a sigmoid curve using.

### Assessment of *C. elegans* larval viability

After 72 hours exposure to 2-isopropyl-5-methylcyclohexanol the *C. elegans* larvae were treated with the fluorometric markers propidium iodide (Invitrogen) or Sytox (Invitrogen) and observed in a fluorescence microscope in order to verify the larvae viability. These markers were used at the following concentrations: 5.0 μmolL^−1^ and 20.0 μmolL^−1^ of Sytox and propidium iodide, respectively [17,18]. Images were taken at microscope (Leica DM500) under 100× magnification; excitation at 510–560 nm and emission at 590 nm for propidium iodide, excitation at 450–490 nm and emission at 535 nm for Sytox. The capture system used was Canon EOS 600D.

## Results

Initially, the crude leaf extract of the of *A. crassiflora* was analyzed by ^1^H NMR and characteristic amino acid and organic acid signals were observed in the *δ* 0.80 to 4.00 region. Most of the signals ranging from *δ* 4.00 to *δ* 5.50 were attributed to the anomeric protons of carbohydrates, and signals at *δ* 5.50 to 8.50 to the signals of aromatic compounds. Comparing our NMR data with the data of the ^1^H NMR signals of metabolites available in the literature [11-14], and performing *J*-resolved and COSY analysis, it was possible to identify various compounds in a single analysis (Table 1).

**Table 1:** **Identified metabolites in** ***Annona crassiflora*** **extracts**

**Substances**	**Chemical shift (** ***δ*** **); multiplicity; [coupling constant (** ***J*** **/Hz)]**
Alanine	1.48; d; [7.2]
Threonine	1.32; d; [6.6]
Valine	1.00; d. [7.0]; 1.05; d; [7.0]; 2.3; m
Choline	3.24; s
Sacarose	5.40; d; [3.8]; 4.17; d; [8.5]
α -glucose	5.18; d; [3.8]
β -glucose	4.58; d; [7.8]
Ferulic acid	7.56; d; [15.9]; 7.19; d; [2.1]; 7.10; dd; [8.4 and 2.1]; 6.88; d; [8.4]; 7.15; d; [2.8]; 6.33; d; [15.9]
Formic acid	8.46; s
γ - aminobutyricacid (GABA)	2.30; t; [7.2]; 3.01. t; [7.5]
Quercetin	7.70; d; [2.0]; 7.66; dd; [8.6 and 2.0]; 6.99; d; [8.6]; 6.52; d; [2.0]; 6.32; d; [1.8]
Trigonelline	9.14; s; 8.87; m

Hydroalcoholic extract activity was observed in the *Caenorhabditis elegans* mobility test, (Table 2). As trigonelline was identified in the hydroalcoholic extract, and it is well known that trigonelline plays an important role in the resistance process of plants against several pathogens [19], commercial standard trigonelline was tested in the same assay. No significant reduction in the mobility of *Caenorhabditis elegans* larvae was observed for trigonelline. From this result, the extract was subjected to partition with CH_2_Cl_2_, ethyl acetate, MeOH and H_2_O, which were submitted to biological evaluation. It was observed that activity was concentrated in the ethyl acetate and dichloromethane fraction (Table 2).

**Table 2:** **Percentage of** ***Caenorhabditis elegans*** **larvae paralyzed after exposure to** ***A. crassiflora*** **extracts**

**Extract, fractions and compounds (1000 μg.mL** ^**−1**^ **)**	**Immobile nematodes (%)** ^***a***^
Hydroalcoholic extract	78.56 d
Aqueous fraction	66.16 c
Methanolic fraction	43.53 b
Ethylacetate fraction	89.66 e
Dichloromethane fraction	98.13 e
Trigonelline	10.06 a
Negative control (DMSO 1% v/v)	4.66 a
Positive control (Ivermectin)	100.00 e

^*a*^Means followed with the same letter do not differ significantly according to the Scott-Knott test (*P* ≤ 0.05).

Dichloromethane and ethyl acetate fractions were analyzed by TLC and ^1^H NMR, which indicated a similar metabolic profile. As a result, these fractions were combined and fractionated in SiO_2_ column, from which were obtained the flavonoids quercetin and kaempferol. The latter had their structures confirmed by comparison of the ^1^H-NMR data with data obtained in the literature [20-23]. In addition, an oil was obtained and was analyzed by gas chromatography–mass spectrometry (GC-MS) and one and two-dimensional NMR. Methyl palmitate ester was identified as the major compound, as well as some minor compounds identified by the NIST libraries, considering the compounds with similarity index equal to or greater than 85% (Table 3).

**Table 3:** **Chemical composition of the oil isolated from** ***Annona crassiflora*** **leaves**

**Compounds**	**Retention time (min)**	**Percentage (%)**
Palmitic acid methyl esther (1)	6.24	48.14
2-isopropyl-5-methylcyclohexanol (2)	6.56	2.39
*γ*-Dodelactone (3)	6.72	3.94
Palmitic acid ethyl esther (4)	6.83	5.20
Oleic acid, methyl esther (5)	7.75	7.74
Stearic acid methyl esther (6)	7.99	11.60

**Figure 1:**
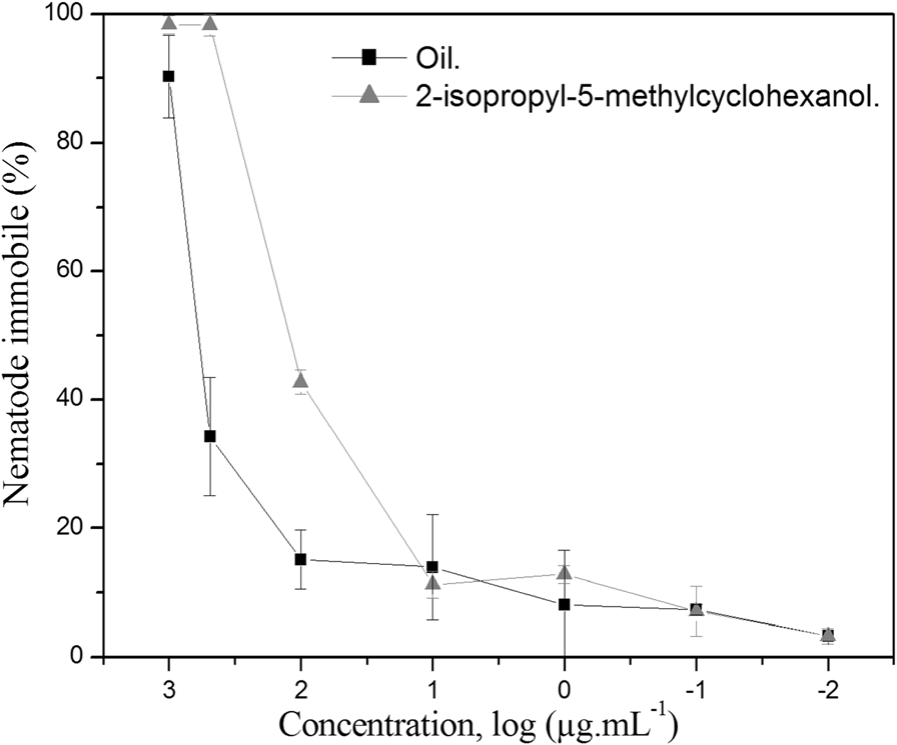
**Effect of oil isolated from the leaves of** ***Annona crassiflora*** **and 2-isopropyl-5-methylcyclohexanol on mobility of** ***Caenorhabditis elegans.***

The oil and the three methyl esthers identified, as well as the 2-isopropyl-5-methylcyclohexanol were evaluated through the mobility test with *C. elegans*. The results indicated that the larvae mobility percentage was directly proportional to the oil and 2-isopropyl-5-methylcyclohexanol concentration (Figure 1). The ED_50_ values, after 72 hours, for oil and 2-isopropyl-5-methylcyclohexanol were 350 and 113 μg.mL^−1^, respectively. The three methyl esters did not show any significant activity.

**Figure 2:**
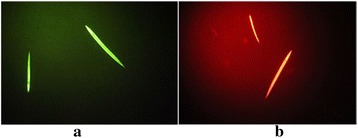
***C. elegans*** **larvae treated with 2-isopropyl-5-methylcyclohexanol, after a period of 72 hours were (a) treated with Sytox (b) and treated with propidium iodide.**

The *C. elegans* larvae treated with 2-isopropyl-5-methylcyclohexanol after 72 hour exposure to the substance at concentration 1000 μg.mL^−1^, were efficiently stained with propidium iodide and Sytox (Figure 2).

## Discussion

Different species of *Annona* have shown nematicidal effects. In *in vitro* assay it was observed that the aqueous extract of *Annona senegalensis* caused a significant reduction in the development of *Haemonchus contortus* nematode eggs [5]. Dang *et al.*, [6] also confirmed the nematicidal effect of methanolic and ethyl acetate extracts of *Annona squamosa* seeds against the plant-parasitic nematodes *Meloidogyne incognita* and *Bursaphelenchu sxylophilus*. In a recent paper, Ferreira and colleagues [7] demonstrated the efficacy of the aqueous extract of *Annona muricata* against eggs, larvae and adult worms of *Haemonchus contortus*. The present study revealed that the *Annona crassiflora* leaf extract, dichloromethane and ethyl acetate fractions showed high activity against *C. elegans* when compared with the synthetic positive control. This potent nematicidal action observed in the hydroalcoholic extract could be due to the known potent nematicidal compounds present in it. Important metabolites are formic acid, toxic on nematodes exposed [24], and γ-aminobutyric acid, which can cause paralysis of nematode muscles [25]. Trigonelline, also identified in the extract, plays an important role in plant resistance to pathogen attack. It is also worth noting the inhibitory activity of trigonelline against enzyme acetylcholinesterase [26], an enzyme that acts on termination of nerve impulses and is the target of several commercial nematicides. Thus, a positive result for trigonelline in *C. elegans* mobility test was expected. However, in the present study this compound was not active against this nematode (Table 2).

The chemical characterization of the isolated oil from the dichloromethane and ethyl acetate fractions revealed the presence of several compounds of fatty nature, being methyl palmitate the major compound. Gu and colleagues [27] reported no effect of methyl palmitate and ethyl stearate, at a concentration of 150 μg.mL^−1^, on the mobility of *C. elegans*. In our bioassays the methyl palmitate enriched oil fraction caused immobility of nematodes at an ED_50_ of 350 μg.mL^−1^. Thus, it is possible that the minor compounds of the oil can induce the nematicidal activity observed. In fact, studies with *C. elegans* have demonstrated that 2-isopropyl-5-methylcyclohexanol and other monoterpenes have nematicidal activity superior to the commercial nematicide Oxamyl [28]. Furthermore, the analysis of staining test with propidium iodide and Sytox suggest that not only 2-isopropyl-5-methylcyclohexanol causes a reduction in larvae mobility but also causes damage to cell membrane integrity and interferes in the cellular process of exogenous molecule exclusion. Both the utilized markers are nucleic acid markers, Sytox is not able to cross plasma membrane of viable cells and propidium iodide is capable of going through intact cell membranes, however it is expelled by cells that have a viable exogenous molecule excretion mechanism [17,18].

## Conclusions

This is the first report of nematicidal activity of *A. crassiflora* leaf ethanolic extract and its fractions. The results revealed this species as a promising source for the discovery of new bioactive compounds against nematodes and its hydroalcoholic extract can also be used in a formulation in order to reduce the intensive use of synthetic nematicides, thus reducing the risks to humans and to the environment.
